# Retrieval augmented generation based dynamic prompting for few-shot biomedical named entity recognition using large language models

**DOI:** 10.21203/rs.3.rs-7216581/v1

**Published:** 2025-08-25

**Authors:** Yao Ge, Sudeshna Das, Yuting Guo, Abeed Sarker

**Affiliations:** 1 Department of Biomedical Informatics, School of Medicine, Emory University, Atlanta, GA, USA; 2 Department of Computer Science, Emory University, Atlanta, GA, USA; 3 National Library of Medicine, National Institutes of Health, Bethesda, MD; 4 Department of Biomedical Engineering, Georgia Institute of Technology and Emory University, Atlanta, GA, USA

## Abstract

Biomedical named entity recognition (NER) is a high-utility natural language processing (NLP) task, and large language models (LLMs) show promise particularly in few-shot settings (i.e., limited training data). In this article, we address the performance challenges of LLMs for few-shot biomedical NER by investigating a dynamic prompting strategy involving retrieval-augmented generation (RAG). In our approach, the annotated in-context learning examples are selected based on their similarities with the input texts, and the prompt is dynamically updated for each instance during inference. We implemented and optimized static and dynamic prompt engineering techniques and evaluated them on five biomedical NER datasets. Static prompting with structured components increased average F_1_-scores by 12% for GPT-4, and 11% for GPT-3.5 and LLaMA 3-70B, relative to basic static prompting. Dynamic prompting further improved performance, with TF-IDF and SBERT retrieval methods yielding the best results, improving average F_1_-scores by 7.3% and 5.6% in 5-shot and 10-shot settings, respectively. These findings highlight the utility of contextually adaptive prompts via RAG for biomedical NER.

## Introduction

Named entity recognition (NER) is a fundamental natural language processing (NLP) task, involving the extraction of predefined entities from free text, with a wide range of application in digital medicine. Within the broader biomedical domain, NER methods can be applied to detect entities such as diseases, adverse drug reactions, treatments, and symptoms. Biomedical NER often presents unique challenges due to the sparsity of certain medical concepts (*e.g*., rare health conditions), and the specialized language used in clinical and social health contexts. While some deep neural network based NER methods have achieved human-like performance, they require large amounts of training data, which is often not available for specialized biomedical problems. Furthermore, NER systems trained on open domain (as opposed to restricted domain) data are typically not transferrable to biomedical domain-specific tasks without additional training or manual annotation. Manually annotating data for targeted biomedical NLP problems is expensive (*e.g*., when they involve medical experts) or infeasible (*e.g*., for sparsely-occurring medical conditions). Even when studies invest time in annotating biomedical data, the datasets cannot be publicly shared (*e.g*., electronic health record data for privacy compliance). Thus, there is a need to develop few-shot learning (FSL) based NER solutions that can generalize effectively from a small number of examples^1^. In this paper, we describe the development and evaluation of an NER approach that leverages a large language model (LLM) and retrieval augmented generation (RAG) for improving the state of the art in few-shot biomedical NER.

The emergence of generative LLMs such as the GPT and LLaMA series has enabled the research community to achieve large performance gains in FSL-based NLP methods. Generic LLMs are adaptable to biomedical domain NLP tasks in zero-shot and few-shot settings^2^, and their ability to adapt to new tasks with minimal examples has been transformative, particularly for restricted domains such as biomedical. By leveraging in-context learning (*i.e*., training examples are provided as part of the prompt), current state-of-the-art LLMs can readily adapt to diverse text structures and vocabularies^3^. A major focus of recent biomedical NLP research has been prompt engineering methods^4,5^, as carefully designed prompts can help align an LLM’s understanding and outputs with task-specific requirements.

The typical approach in prompt-driven methods is to optimize predefined,*static* prompts for a target task. Static, in this context, refers to the use of the same, consistent prompt for every instance in a dataset during inference. Thus, regardless of the content of the input text, the model applies the same fixed prompt and in-context examples. This lack of flexibility to adjust to specific input data potentially leads to sub-optimal performance^6^. Their fixed format restricts performance potential, as they do not adjust based on context, even when more suitable annotated examples are present in the training data. Consequently, approaches employing static prompts exhibit high variance depending on the relevance of the in-context examples to the unlabeled input texts^7^.

More sophisticated architectures include approaches such as retrieval-augmented generation (RAG) and chain-of-thought (CoT) prompting^8^. CoT is a reasoning paradigm that enables models to generate intermediate reasoning steps, mimicking human-like problem-solving processes^9^. It enhances performance on complex tasks by decomposing problems into sequential, logical steps, improving both accuracy and interpretability in multi-step reasoning scenarios. RAG involves retrieving query-relevant information, and enriching the context of the LLMs with retrieved contents prior to generation^10^. The retrieval process is typically guided by similarity measures^11^, such as cosine similarity between embeddings, which helps the model access contextually relevant examples or documents tailored to the input query. Once these relevant texts are retrieved, they are integrated into the prompt or used as additional context to aid the model’s response generation, resulting in a more contextually informed output.

The motivation behind RAG is to address the limitations of LLMs in handling tasks that require specialized or up-to-date information^12^. Even with extensive training data, LLMs may struggle with domain-specific concepts or recent developments due to knowledge cutoffs and lack of domain specificity in training corpora^13^. By introducing contextually relevant information at inference time, RAG can significantly improve performance in specialized applications^14^, such as biomedical text analysis, where precision and relevance are critical. In biomedical NER, RAG can improve the adaptability of a model by retrieving examples or contexts that closely resemble the input text, thereby increasing the identification accuracy of entities^14^. In FSL settings, RAG architectures have the potential to reduce reliance on large annotated datasets by dynamically selecting relevant data^15^, making it particularly useful for domains where annotated data is limited. Additionally, RAG complements prompting techniques, like CoT prompting, by enabling stepwise reasoning based on retrieved information^16^, which may lead to better precision and recall for complex, sparse entities.

With the aim of addressing the inherent limitations of static prompts and improving biomedical NER performance in FSL settings, we explore dynamic prompting techniques, which involve automatically retrieving suitable training examples and adjusting prompts based on contextual similarity. Following the optimization of prompts, we evaluate the effectiveness of the two types of prompting—static and dynamic—using three LLMs: GPT-3.5, GPT-4, and LLaMA 3 (open source), on five datasets. We investigate the effectiveness of both static and dynamic prompt engineering strategies, integrated with retrieval mechanisms, to improve few-shot NER. We systematically evaluate multiple retrieval mechanisms and investigate how prompt design choices affect model performance. Our results demonstrate the potential of these techniques to enhance entity recall and precision in biomedical NER, offering insights into the optimization of LLMs for biomedical applications.

The primary contributions of this work are as follows:

- Development of a structured static prompt optimization framework, incorporating task-relevant instructions, entity definitions, and dataset contextualization to improve few-shot biomedical NER.
- Integration and evaluation of RAG, assessing how distinct retrieval mechanisms (TF-IDF, SBERT, ColBERT, and DPR) enhance dynamic prompting by selecting contextually relevant examples.
- Comparative analysis of static and dynamic prompting strategies, benchmarking their effectiveness in few-shot biomedical NER, and offering insights into their strengths across different datasets.

## Results

### Task-specific Static Prompting

The results in Table 1 demonstrate consistent performance improvements across the five biomedical datasets when multiple components of the static prompting strategy are combined for all three LLMs. Compared to the baseline prompt, the addition of task-specific components, such as dataset descriptions, high-frequency instances, error analysis, and few-shot examples, led to significant improvements in precision, recall, and F_1_-score across all datasets. GPT-4 showed the largest improvements when the full structured prompt was used. For GPT-4, the average F_1_-score increased by 12.0% across datasets, ranging from 6.95% for MIMIC III to 23.7% for Med-Mentions. GPT-3.5 obtained an average F_1_-score increase of 11.4%, with gains ranging from 7.1% for BC5CDR to 22.9% for Med-Mentions. LLaMA 3-70B, which started with the lowest baseline performance, showed an average F_1_-score increase of 11.1%, with its largest improvement also observed in the Med-Mentions dataset (21.44%).

GPT-4 consistently outperformed GPT-3.5 and LLaMA 3-70B in all configurations, benefiting more from the integration of task-specific components, particularly in datasets such as BC5CDR and Med-Mentions, where it achieved the highest F_1_-score. GPT-3.5 and LLaMA 3, while achieving slightly lower overall performance still exhibited performance improvements relative to the baseline. This is evident in datasets such as Reddit-Impacts, where its F_1_-score exhibited significant improvement with the integration of additional components.

As illustrated in Table 1, high-frequency instances (see Section Static Prompt Engineering), and dataset descriptions had the most notable impact on recall. For example, in the Med-Mentions dataset, adding high-frequency instances improved recall for GPT-4 by 6.57%. Few-shot examples at the token level provided the most significant increase in precision across models. For instance, precision in the NCBI dataset increased by 21.9% for GPT-3.5 and by 15.6% for GPT-4.

**Figure 1.**
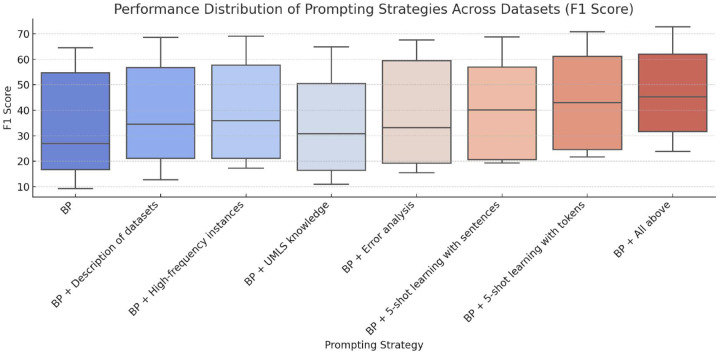
Performance distribution of prompting strategies across datasets (F_1_-score). The box plots depict the performance of various prompting strategies applied to five biomedical datasets, highlighting the range, median, and distribution of F_1_-scores for each strategy.

Figure 1 highlights the performance changes associated with different prompting strategies across datasets. The incorporation of knowledge from the unified medical language system (UMLS) improved recall in certain datasets such as BC5CDR but underperformed compared to the baseline prompt for datasets like Reddit-Impacts and NCBI. This component aimed to provide foundational biomedical knowledge by introducing descriptions and context derived from UMLS. However, this approach may have introduced noise, particularly in datasets that are not strongly aligned with UMLS’s predefined biomedical concepts. For example, in the Reddit-Impacts dataset, GPT-3.5’s F_1_-score decreased slightly from 16.7 to 16.4, suggesting that the background information from UMLS diluted the model’s ability to capture task-specific cues. 95% confidence intervals (CIs) for each metric are provided in Table 6 in Supplementary Materials.

### Dynamic Prompting with RAG

The results in Table 2 demonstrate the effectiveness of dynamic prompting in multiple FSL settings (5-shot, 10-shot, and 20-shot) for GPT-4 and LLaMA 3 across five biomedical datasets*. As described in the Section Experimental Setup, the baseline prompts used randomly selected examples, and the results were averaged over four random runs. Detailed results for each random run, along with the averaged results, are presented in Table 1 to Table 5 in Supplementary Materials. 95% confidence intervals (CIs) for each metric are provided in Table 7 in Supplementary Materials. We also provided examples of predictions in Supplementary Materials.

Both LLMs benefit significantly from retrieval-augmented methods. TF-IDF and ColBERT frequently produce the highest F_1_-scores for both models. SBERT also consistently improves over the base method, especially for GPT-4. For GPT-4, TF-IDF retrieval outperforms other methods in most cases. For example, on the BC5CDR dataset, TF-IDF achieves the highest F_1_-score of 85.9% in the 5-shot setting, 86.6% in the 10-shot setting, and 87.2% in the 20-shot setting. Similarly, for the MIMIC III dataset, TF-IDF achieves the top F_1_-score of 76.2% in the 5-shot setting and 77.7% in the 20-shot setting. In contrast, SBERT exhibits strong performance on the Reddit-Impacts dataset, where it achieves the highest F_1_-scores of 33.7% (5-shot) and 35.5% (10-shot). Moreover, SBERT achieves an F_1_-score of 41.43% on REDDIT-IMPACTS in the 20-shot setting, outperforming TF-IDF by a margin of 3.08%. For LLaMA 3, DPR retrieval achieves competitive results, particularly on BC5CDR, where it achieves the highest F_1_-scores of 84.8% (10-shot) and 74.8% (20-shot). SBERT also performs strongly on the REDDIT-IMPACTS dataset, achieving F_1_-scores of 34.4% (5-shot) and 41.4% (20-shot).

**Figure 2.**
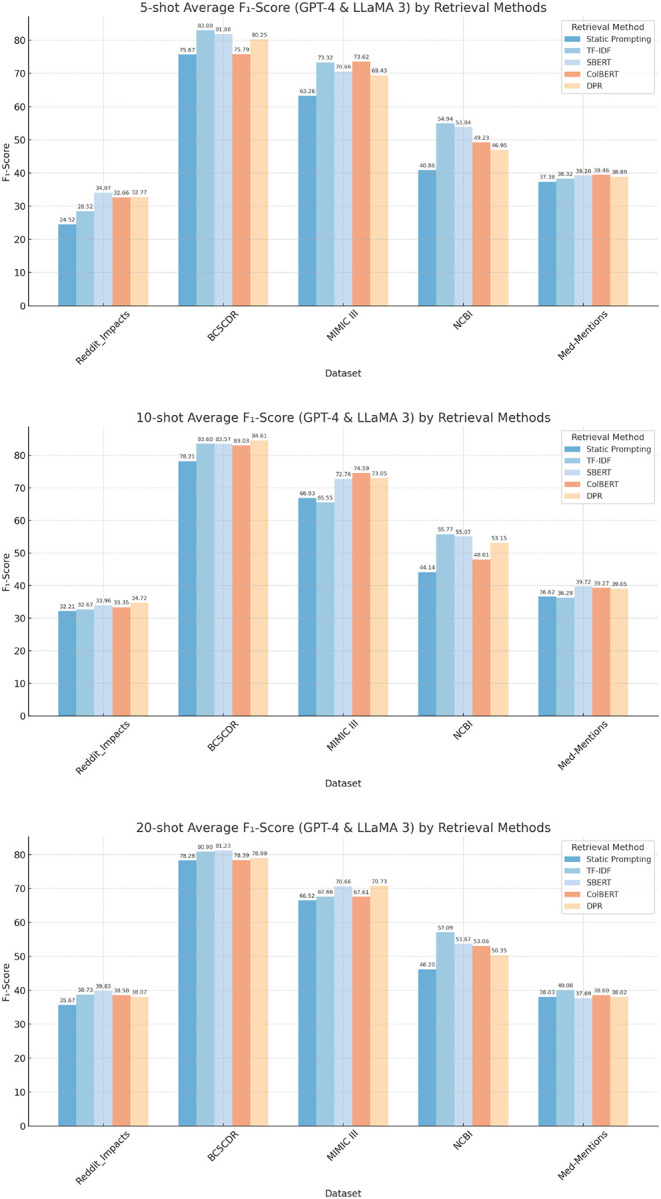
Comparison of average F_1_-scores for GPT-4 and LLaMA 3 models across different datasets under varying shot settings.

Figure 2 presents the F_1_-scores of five biomedical datasets by using different retrieval methods compared to static prompting for multiple shot settings: 5-shot, 10-shot, and 20-shot. The results are averaged across evaluations conducted using GPT-4 and LLaMA 3 models. Across all settings, all retrieval-based methods show significant improvements, demonstrating the benefit of incorporating retrieval methods into the prompting strategy.

#### 1. 5-shot Analysis:

The SBERT retrieval engine achieves the highest average F_1_-score for the Reddit-Impacts dataset (34.1%), while TF-IDF performs best on BC5CDR (83.0%) and NCBI (54.9%). For MIMIC III, ColBERT leads with an F_1_-score of 73.6%, and on Med-Mentions, ColBERT also stands out with a top score of 39.5%. TF-IDF achieved an average F_1_-score improvement of 7.28% across all datasets in the 5-shot setting, followed closely by SBERT with 7.46%. ColBERT and DPR showed more modest improvements, with 5.76% and 4.81%, respectively. These results highlight the dataset-specific strengths of different retrieval methods, with TF-IDF showing strong performance on entity-rich datasets like BC5CDR and NCBI.

#### 2. 10-shot Analysis:

The SBERT engine again stands out as the best-performing retrieval method overall, achieving the highest F_1_-scores on three datasets: Reddit-Impacts (34.0%), Med-Mentions (39.7%), and NCBI (56.1%). DPR achieves the top score on BC5CDR (84.8%), while ColBERT performs best on MIMIC III with an F_1_-score of 74.6%. Consistent with these findings, SBERT also demonstrates the largest overall improvement over the base method, with an average gain of 5.59%. It is followed by ColBERT with 4.87% and DPR with 4.50%, while TF-IDF shows the smallest improvement in the 10-shot setting, with an average increase of 3.49%. These results highlight a departure from the 5-shot setting, where TF-IDF dominated, indicating that SBERT is better suited for slightly larger data scenarios.

#### 3. 20-shot Analysis:

TF-IDF once again demonstrates strong performance, achieving the highest F_1_-scores on three datasets: BC5CDR (82.8%), NCBI (55.9%), and Med-Mentions (40.1%). SBERT leads on Reddit-Impacts with a top score of 39.8%, while it also performs best on MIMIC III with an F_1_-score of 70.2%. Among the retrieval approaches, in the 20-shot setting, TF-IDF achieves the highest average improvement with 3.96%, followed closely by SBERT with 3.55%. DPR shows a moderate improvement with 2.08%, while ColBERT exhibits the lowest increase of 1.95%. These results highlight TF-IDF’s and SBERT’s consistent robustness across multiple datasets as the top-performing retrieval method.

Overall, GPT-4 consistently achieves higher F_1_-scores compared to LLaMA 3 across most datasets and retrieval methods, particularly in 5-shot setting, with an average F_1_ score 17.3% higher than LLaMA 3. In the 10-shot setting, this gap narrows to 5.47%, but GPT-4 still maintains a clear advantage. Finally, in the 20-shot setting, GPT-4 surpasses LLaMA 3 by an average F_1_ score of 8.30%. This improvement becomes even more significant in datasets with sparse or noisy data, where retrieval-augmented methods play a critical role. LLaMA 3 shows comparable performance in 20-shot setting but struggles to close the gap with GPT-4 in scenarios with fewer examples or more noisy data. This highlights GPT-4’s robustness in leveraging limited training data.

Across all datasets and shot settings on GPT 4, larger training sizes (20-shot) tend to yield higher F_1_-scores, precision, and recall. Specifically, from 5-shot to 10-shot, the mean F_1_-score increases by 2.51%, while precision and recall improve by 2.22% and 1.02%, respectively. However, from 10-shot to 20-shot, the performance gains are notably smaller, with F_1_-score increasing by 1.64%, precision by 0.16%, and recall by only 0.05%. Overall, comparing 5-shot to 20-shot, the models achieve a cumulative improvement of 4.16% in F_1_-score, 2.38% in precision, and 1.07% in recall. However, on LLaMA 3, this increase is less consistent, with the best performance observed at the 10-shot setting across all datasets except for the Reddit-Impacts dataset. From 10-shot to 20-shot, mean F_1_-score decreases by −1.46%, mean precision drops by −1.36%, and mean recall declines by −1.75%. The combination of effective retrieval methods and larger shot sizes (more examples) contributes significantly to the overall improvements observed in model performance across all datasets.

## Discussion

NER is one of the most commonly applied NLP tasks, and while the emergence of LLMs have led to substantial leaps in few-shot NER performance, innovative strategies are needed to address some of their limitations for real-life application in biomedicine. Improving FSL NER methods involving LLMs has the potential to substantially reduce the time and cost required for manual annotation. The methods proposed in this paper, validated on multiple standardized datasets with differing characteristics, present an important step towards operationalizing automated NER from biomedical texts, including in healthcare settings.

Our extensive empirical explorations revealed findings that will be useful for future research and application in this space. First, GPT-4 consistently outperforms GPT-3.5 and LLaMA 3-70B across datasets and configurations, demonstrating its robustness in understanding nuanced biomedical information. The consistent high performance of GPT-4 may be attributable to several factors. GPT-4 has significantly more parameters compared to GPT-3.5 and LLaMA 3-70B, enabling it to capture finer-grained contextual nuances, especially in complex and domain-specific tasks. Furthermore, in datasets with sparse or ambiguous annotations, such as Reddit-Impacts or Med-Mentions, GPT-4 achieves higher recall, indicating its ability to identify relevant entities and relationships more comprehensively. Further, retrieval engines improve performance by providing task-relevant context that enhances the model’s understanding of the input, effectively bridging the gap between the model’s general pretraining knowledge and the specific requirements of the task. Our results broadly show that TF-IDF based retrieval works well for datasets that have low noise and limited out-of-vocabulary expressions, despite its simplicity. In contrast, engines like SBERT perform better on linguistically diverse datasets, especially on Reddit-Impacts dataset, by leveraging semantic embeddings, which capture nuanced relationships between words and phrases. Advanced retrieval methods like ColBERT and DPR generally underperformed compared to TF-IDF and SBERT. This may be due to several reasons. ColBERT and DPR rely on dense representations, which, while powerful for general-purpose semantic matching, may fail to capture the precise, domain-specific distinctions critical in biomedical datasets. Furthermore, their reliance on dense embeddings can sometimes overfit to irrelevant semantic similarities, retrieving documents that are semantically related but not contextually relevant to the query.

Given these findings, our results suggest that TF-IDF is the most efficient option for retrieval in datasets with low noise, while SBERT is better suited for handling linguistically diverse data. ColBERT and DPR, despite their strengths in general-purpose retrieval, do not provide substantial advantages in this domain and may introduce unnecessary computational overhead. Thus, for biomedical applications requiring high precision and efficiency, TF-IDF and SBERT offer the best balance of performance and efficiency. The effect of shot size on performance is not uniform, as observed in the results across datasets. While increasing the shot size from 5 to 20 generally improves F_1_-scores, the extent of improvement is dataset-dependent. Datasets with formal texts, like BC5CDR, which already benefit from the inclusion of the retrieval engine, exhibit marginal gains with additional examples. In contrast, noisy datasets like Reddit-Impacts are more sensitive to shot size, as more examples help the model adapt to diverse linguistic patterns and reduce misclassifications. 20-shot does not always yield the best results. One reason is diminishing returns: as the number of examples increases, redundancy or noise may be introduced, especially in datasets where retrieval engines already provide strong task-specific context. Another potential reason arises from the inherent constraints of LLMs, such as input token limits. As the shot size grows, the available space for processing task-specific context diminishes, potentially diluting the effectiveness of the prompt or truncating important information. Our findings suggest that for NER tasks involving sparsely-occurring entities, RAG-based dynamic prompting is likely to obtain better performance compared to optimized, static prompts. For retrieval, performances of the different engines were mostly comparable, and TF-IDF and SBERT consistently performed well. As the number of training instances increased, the impact of dynamic prompting over static prompting (Base), became less visible. This is expected since in high-shot settings, random draws of training instances contain considerable diversity to enable model generalization. It is also possible that as the number of examples provided for in-context learning increases, the overall increase in the length of the prompt diminishes the performance of the LLMs. The influence of input text length and LLM performance is an area of active research^17^.

## Methods

### Static Prompt Engineering

**Figure 3.**
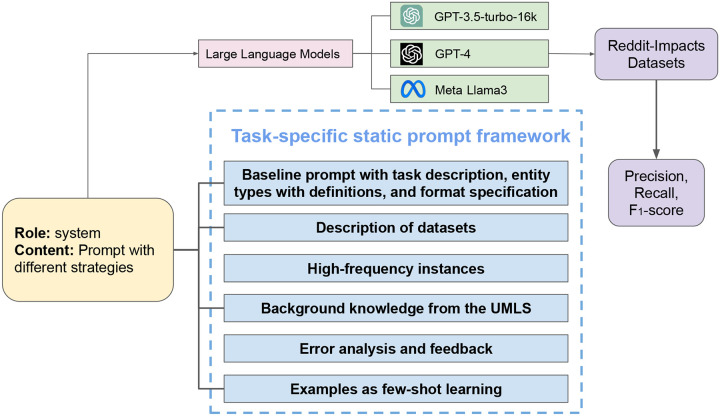
An overview of the NER strategy based on static prompting on three LLMs. Static prompts containing different information are provided to the LLMs, which, in turn, generate predictions for evaluation.

Figure 3 presents the components of the static prompt we optimized for the LLMs. We systematically designed task-specific static prompts comprising the following components:

#### 1. Baseline prompt with task description, entity types with definitions, and format specification:

The baseline component provides the LLM with essential information regarding the primary aims of the task, which is extracting and classifying entities. The categories of labels present in the dataset, along with their definitions. Entity definitions provide detailed and unequivocal explanations of an entity in the context of a specific task, crucially guiding the LLM toward accurately pinpointing entities within texts. Also, we provided the input, and instructions regarding the output format in the base prompt. For generative LLMs, NER presents greater challenges, relative to classification, as it is essentially a sequence-to-sequence problem, where each token is assigned a corresponding label. However, when a prompt includes a sentence as is, we found that LLMs may struggle to accurately assign labels to each token, resulting in mismatches in the number of input tokens (as annotated in the dataset) and output tokens. This issue is exacerbated by the fact that LLMs have their own tokenization mechanisms, which may differ from the tokenization in the annotated data. If the input and labels are provided in the BIO format instead, it often results in degraded performance due to the LLM’s inability to fully understand the text.

One input approach is to provide a text and indicate the entities within it^18^. For example, in the sentence ’I was a codeine addict,’ the phrase ’codeine addict’ is identified as an entity and is annotated as ‘Clinical Impacts’. However, this format can become ambiguous when faced with long sentences that contain the same word or phrase multiple times, each with different contextual meanings, not all of which may be labeled as the relevant entity. Another input method involves providing spans corresponding to the entities^19^, but this also causes mismatches between spans and entities frequently when generative LLMs are used.

To address these challenges, we adopt a new format for constructing the input and output for the LLMs. We provide LLMs with a list of tokens that have already been tokenized. For the output, we instruct the model to return each token, concatenated with its corresponding label. This method allows us to easily extract labels for evaluation, and it ensures a one-to-one correspondence between the predicted labels and tokens, with the number of labels always consistent with the number of tokens in the input sentence.

For example:

Input: [‘I’, ‘was’, ‘a’, ‘codeine’, ‘addict.’]
Output: [‘I-O’, ‘was-O’, ‘a-O’, ‘codeine-B-Clinical_Impacts’, ‘addict.I-Clinical_Impacts’]

To minimize the potential loss of sentence context caused using only tokens, we also explored the effectiveness of using the untokenized sentences as input, and tagged tokens as output:

Input: [‘I was a codeine addict.’]
Output: [‘I-O’, ‘was-O’, ‘a-O’, ‘codeine-B-Clinical_Impacts’, ‘addict.I-Clinical_Impacts’]

#### 2. Description of datasets:

By describing a dataset’s origin, content, and themes, we aim to provide LLMs with a basic understanding of the dataset. For example, for the Reddit-Impacts dataset, we described that it focuses on individuals who use opioids, and we are interested in the impact of opioid use on their health and lives.

#### 3. High-frequency instances:

Some entities do not have clear definitions, and the determination is more ambiguous. Therefore, we provide the most frequently occurring words or phrases in each entity type within the training dataset to assist LLMs in understanding the potential distribution of entities and the theme of the text for this task. Specifically, we selected high-frequency instances for each class by computing word frequencies from lexicon-annotated data, ranking them by occurrence, and choosing the top 6 high-frequency words as label words for each class. This approach ensures that the selected label words effectively reflect the data distribution and help the model predict appropriate class labels at entity positions. By adding high-frequency instances, we tried to provide a LLM with a lexicon of the concepts of interest.

#### 4. Incorporation of background knowledge from the UMLS:

We provide LLMs with comprehensive and structured information we obtained from the UMLS. Our intuition, based on the findings reported in prior work, was that this knowledge could enhance the understanding and interpretation of biomedical concepts, relationships, and terminologies.

#### 5. Error analysis and feedback:

To improve the model’s accuracy and address prediction errors, we provide an error analysis and feedback mechanism. After an initial set of predictions was made by LLMs on unseen training set instances, we manually reviewed the errors by comparing the model’s predictions with the gold standard annotations. For each incorrect prediction, we analyze the type and cause of the error, such as misclassification, missed entities, or spurious entities. Based on this analysis, we provide a summarization of feedback to the model. This feedback includes only general descriptions of errors without any examples. While this element of the prompt requires preliminary explorations of the dataset, common possible errors can be identified easily using a small set of training examples (*e.g.,* 5-shot), and this enables a mechanism of incorporating expert feedback into the process.

#### 6. Annotated samples:

We provide *k* annotated instances within the prompt for in-context learning. Samples are randomly selected and formatted according to the task description and entity markup guide.

We compared the effectiveness of different components of static prompting by incrementally incorporating descriptions of datasets, high-frequency instances, background knowledge from the UMLS, error analysis and feedback, and varying k-shot annotated samples. Detailed prompts used for each dataset are provided in Table 8 to Table 12 in Supplementary Materials.

### Dynamic Prompt Engineering

In prompt-based strategies using LLMs for in-context learning, the common approach has been to provide the model with a static prompt to guide its predictions. These prompts often include example instances, and CoT prompting. However, a significant limitation of this approach is that the provided examples may differ substantially from the texts from which the model is expected to extract named entities. Note that even in the presence of additional annotated samples, the LLMs context window size may limit the number of instances that can be embedded in a prompt for in-context learning. A static prompt, thus, does not generalize well, leading to high variance in performance.

**Figure 4.**
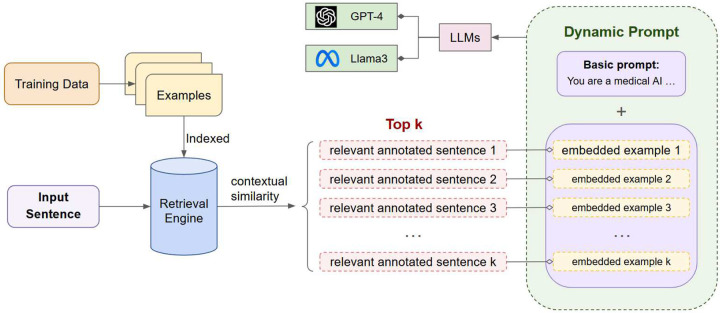
Overview of Retrieval-based Dynamic Prompting model. First the training data are provided to the retrieval engine for indexing. During inference, the system first ranks all training examples based on contextual similarity with the input text. Finally, the top *n* retrieved instances are embedded in the prompt, which is passed to the LLM (e.g., GPT-4, LLaMA 3).

To address this issue, we attempted to improve upon static prompting and adopted a dynamic approach involving RAG. In our proposed approach, a retrieval engine is first indexed with the annotated examples from the training set. Upon receiving an input sentence, the system first retrieves the top *n* annotated examples using the retrieval engine. The retrieved examples are then embedded into the prompt, which is then passed to the LLM along with the input text. Figure 4 presents an overview of the system architecture.

### Retrieval Engines

Selecting an effective retrieval engine is crucial since the examples embedded in the prompt influence the model’s performance. We considered several retrieval methods, each chosen for its unique strengths in handling diverse biomedical texts, and applicability in FSL settings. The engines we selected are: TF-IDF^20^, Sentence-BERT (SBERT)^21^, ColBERT^22^, and Dense Passage Retrieval (DPR)^23^. These search mechanisms offer a range of capabilities, from efficient keyword matching to advanced deep-learning-based retrieval. We provide further details below.

#### 1. TF-IDF:

Term Frequency-Inverse Document Frequency (TF-IDF) scores the relevance of documents based on the frequency of terms. We included TF-IDF due to its efficiency and simplicity, which allows for rapid retrieval of relevant examples based on keyword overlap. While it lacks semantic understanding, it serves as a strong baseline, particularly when the input contains well-defined biomedical terminologies.

#### 2. Sentence-BERT (SBERT):

SBERT leverages a pre-trained BERT model fine-tuned for semantic similarity tasks. By encoding input sentences into dense embeddings, SBERT can capture the semantic relationships between sentences, making it well-suited for identifying contextually similar examples even when the input phrasing differs from the training data. This capability is particularly advantageous in the biomedical domain, where synonymous terms and varied expressions are common.

#### 3. ColBERT:

ColBERT (Contextualized Late Interaction over BERT) enhances retrieval performance by focusing on contextualized token representations. It uses a late-interaction mechanism that allows for more nuanced matching of query and document tokens. We selected ColBERT for its ability to capture fine-grained semantic details, which is essential for handling complex biomedical texts with diverse and context-dependent entity mentions.

#### 4. Dense Passage Retrieval (DPR):

DPR employs a dual-encoder architecture, where separate encoders are used for queries and documents. It uses deep neural networks to learn dense embeddings, optimizing for maximum similarity between relevant query-document pairs. DPR’s strength lies in its ability to handle open-domain retrieval tasks effectively, making it a powerful choice for dynamically selecting annotated examples that are highly relevant to the input text, thus improving the contextual adaptability of our dynamic prompts.

In our experiments, we evaluated the performance of each retrieval method, assessing their impact on few-shot NER across multiple biomedical datasets.

### Experimental Setup

Below, we report our experimental setup for the two prompting strategies—Static Prompting and Dynamic Prompting.

#### 1. Static Prompting:

For static prompting, we evaluated three language models: GPT-3.5, GPT-4, and LLaMA 3. We used prompts containing five examples per label to provide context and guide the models’ predictions. For GPT-3.5, we used the OpenAI API version “2023-07-01-preview”, and for GPT-4, we used the version “2024-02-15-preview”. Both models were configured with the following settings: temperature = 0.2, top_p = 0.1, frequency_penalty = 0, presence_penalty = 0, and no stop tokens specified.

For LLaMA 3, we used the Meta-Llama-3-70B-Instruct model, with a temperature of 0.5 and top_p of 0.95. Preliminary experiments (reported later in this chapter) revealed that GPT-3.5 consistently performed significantly worse compared to GPT-4. Hence, we excluded GPT-3.5 from further experiments in the dynamic prompting phase to limit API usage costs. To ensure robustness in the static prompting phase, the few-shot examples were randomly selected four times, and the reported results are the average of these four random selections.

#### 2. Dynamic Prompting:

In the dynamic prompting phase, we focused on evaluating GPT-4 and LLaMA 3 on multiple datasets. We conducted experiments using three different in-context settings: 5-shot, 10-shot, and 20-shot, to assess the impact of increasing the number of examples on the model’s performance. The baseline prompts in this phase also used randomly selected examples, with the results averaged over four random runs.

The evaluations were conducted on five biomedical datasets: MIMIC-III (clinical notes dataset), BC5CDR (disease and chemical entity recognition), NCBI-Disease (disease annotations from PubMed abstracts), Med-Mentions (large-scale UMLS concepts dataset), and our Reddit-Impacts dataset (annotated for clinical and social impacts entity extraction). Further details about these datasets are provided in Chapter 3. We used precision (P), recall (R), and F_1_-score (F_1_) as evaluation metrics to comprehensively asses the models’ performance across different datasets. In addition, to account for the variability in performance across different experimental runs, we include 95% confidence intervals (CIs)^24^ for each metric, providing a measure of the statistical robustness of the results. The confidence intervals were computed via bootstrap resampling^25^ with 1000 samples with replacement.

## Supplementary Material

This is a list of supplementary files associated with this preprint. Click to download.

- RAGbasedDynamicPromptingSupplementaryMaterials.zip

## Figures and Tables

**Table 1.** Performance comparison of various prompting strategies across different datasets in terms of F_1_-score (F_1_), Precision (P), and Recall (R). The row “BP + All components” represents the combination of all strategies, with the best performance across datasets highlighted in bold. The red bold font indicates the best F_1_ score achieved by an individual component, while black bold font highlights the highest Precision, and underlined text denotes the best Recall for a single component. Additionally, green bold font is used to mark F_1_ scores that are lower than the baseline performance (BP).

	*Reddit_Impacts*			*BC5CDR*			*MIMIC III*			*NCBI*			*Med-Mentions*		
	P	R	F_1_	P	R	F_1_	P	R	F_1_	P	R	F_1_	P	R	F_1_
**GPT-3.5**															
**Basic Prompt (BP)**	10.37	43.26	16.73	55.64	76.88	64.56	55.31	54.11	54.70	18.28	51.33	26.96	8.55	10.12	9.27
**BP + Description of datasets**	13.25	52.38	21.15	59.25	81.47	68.61	59.54	54.18	56.73	26.24	50.25	34.48	16.52	10.33	12.71
**BP + High-frequency instances**	13.40	50.13	21.15	59.08	82.96	69.01	59.93	55.66	57.72	26.54	55.68	35.95	20.16	15.03	17.22
**BP + UMLS knowledge**	10.17	42.86	**16.44**	54.24	80.57	64.83	47.16	54.50	**50.57**	23.93	43.02	30.75	10.26	11.58	10.88
**BP + Error analysis**	12.02	48.21	19.24	57.36	82.49	67.67	64.63	55.16	59.52	25.23	48.31	33.15	18.74	13.24	15.52
**BP + 5-shot learning with sentences**	12.33	44.42	19.30	59.30	82.04	68.84	53.09	53.37	57.03	37.09	43.78	40.16	17.28	25.54	20.61
**BP + 5-shot learning with tokens**	**13.63**	53.11	**21.69**	**62.27**	82.02	**70.79**	**67.50**	55.97	**61.21**	**40.15**	46.32	**43.01**	**20.54**	30.57	**24.57**
**BP + All above**	**15.36**	**53.92**	**23.91**	**63.64**	**84.86**	**72.73**	**67.77**	**57.10**	**61.99**	**42.73**	**48.07**	**45.24**	**22.15**	**55.32**	**31.63**
**GPT-4**															
**Basic Prompt (BP)**	12.75	48.15	20.16	59.56	83.22	69.43	57.57	55.72	56.63	25.13	50.48	33.56	18.27	11.12	13.83
**BP + Description of datasets**	15.12	52.94	23.52	60.66	84.58	70.65	63.35	56.42	59.68	26.43	55.22	35.75	21.23	11.96	15.30
**BP + High-frequency instances**	15.98	53.75	24.64	63.89	84.06	72.60	**64.61**	56.14	60.08	35.02	41.44	37.96	21.72	17.69	19.50
**BP + UMLS knowledge**	12.85	50.14	20.46	59.48	84.63	69.86	55.37	54.90	**55.13**	22.80	47.92	**30.90**	18.72	11.83	14.50
**BP + Error analysis**	14.87	52.04	23.13	67.92	82.75	74.61	63.93	56.72	60.11	34.86	41.38	37.84	20.28	16.59	18.25
**BP + 5-shot learning with sentences**	14.71	51.48	22.88	65.04	83.18	73.00	58.49	55.03	58.25	36.96	45.67	40.86	27.42	30.33	28.80
**BP + 5-shot learning with tokens**	**17.23**	52.57	**25.95**	**68.10**	87.66	**76.65**	63.40	62.49	**62.94**	**40.72**	48.42	**44.24**	**27.71**	41.41	**33.20**
**BP + All above**	**18.87**	**52.01**	**27.60**	**68.62**	**90.32**	**78.03**	63.06	**64.12**	**63.58**	**45.02**	49.02	**46.93**	27.26	**60.06**	**37.49**
**Llama3-70B**															
**Basic Prompt (BP)**	9.93	36.42	15.61	52.52	76.04	62.13	46.57	55.64	50.70	15.63	24.37	19.15	19.59	23.17	21.23
**BP + Description of datasets**	13.27	35.26	19.28	58.53	80.22	67.68	56.64	55.81	56.22	17.30	36.43	21.44	23.59	19.87	21.57
**BP + High-frequency instances**	**14.52**	34.53	**20.44**	60.85	78.05	68.39	55.65	56.47	56.06	21.11	42.97	26.62	**23.99**	31.20	27.12
**BP + UMLS knowledge**	7.87	35.88	**12.91**	57.11	74.63	64.71	46.78	51.63	**48.92**	14.95	34.72	20.91	22.09	25.51	23.68
**BP + Error analysis**	12.46	38.86	18.87	58.96	80.52	68.07	63.18	55.20	58.92	16.84	44.65	24.46	22.64	29.92	25.78
**BP + 5-shot learning with sentences**	11.35	39.71	17.65	65.16	77.28	70.70	62.90	51.61	56.85	21.97	49.94	30.52	23.87	64.66	34.87
**BP + 5-shot learning with tokens**	13.33	40.32	20.04	**66.03**	78.57	**71.76**	**63.89**	60.19	**61.98**	**34.49**	32.41	**33.42**	23.72	68.45	**35.23**
**BP + All above**	13.16	**57.86**	**21.43**	**68.97**	78.36	**73.32**	59.30	**67.27**	**62.94**	**35.81**	**34.71**	**34.80**	**25.89**	**67.05**	**37.26**

**Table 2.** Evaluation of dynamic prompting strategies (5-shot, 10-shot, and 20-shot) using GPT-4 and Llama 3 across five biomedical datasets. The table presents F_1_-score, precision, and recall for each retrieval method: Base Prompt, TF-IDF, SBERT, ColBERT, and DPR. The row “Base” represents using static prompts we proposed in the former section.

		*Reddit_Impacts*			*BC5CDR*			*MIMIC III*			*NCBI*			*Med-Mentions*		
		P	R	F_1_	P	R	F_1_	P	R	F_1_	P	R	F_1_	P	R	F_1_
**GPT-4**																
5-shot	**Base**	18.87	52.01	27.60	68.62	90.32	78.03	63.06	64.12	63.58	45.02	49.02	46.93	27.26	60.06	37.49
	**TF-IDF**	19.71	51.25	28.47	**82.31**	89.76	**85.88**	**74.43**	78.14	**76.24**	**56.86**	63.68	**60.08**	27.22	62.68	37.96
	**SBERT**	**24.31**	55.00	**33.72**	76.63	91.41	83.37	72.63	74.27	73.44	55.05	60.30	57.56	28.05	64.65	39.12
	**ColBERT**	22.66	56.79	32.39	78.64	81.03	79.82	74.14	77.02	75.56	50.43	54.48	52.38	**28.14**	68.69	**39.93**
	**DPR**	22.60	58.79	32.64	79.39	88.24	83.58	69.77	70.00	69.89	46.67	52.39	49.37	27.90	65.49	39.13
10-shot	**Base**	22.25	56.66	31.92	75.33	88.31	81.27	66.38	74.24	70.09	53.23	52.13	52.67	26.67	59.20	36.74
	**TF-IDF**	21.53	56.25	31.14	83.81	89.67	**86.64**	73.85	77.29	75.53	**58.81**	65.66	**62.05**	28.14	71.42	40.37
	**SBERT**	**25.41**	58.75	**35.47**	83.94	87.99	85.92	72.73	75.08	73.89	58.79	63.02	60.83	**28.32**	70.26	40.37
	**ColBERT**	23.86	58.02	33.81	83.49	88.05	85.71	**74.69**	78.06	**76.34**	55.12	59.56	57.25	28.15	71.99	**40.48**
	**DPR**	22.96	56.25	32.61	**85.16**	84.42	84.79	71.84	72.42	72.13	56.82	60.72	58.70	28.25	70.04	40.25
20-shot	**Base**	27.74	58.75	37.67	74.57	89.18	81.15	70.65	71.32	70.98	51.68	52.29	51.98	28.10	60.78	38.39
	**TF-IDF**	27.72	62.20	38.35	85.41	88.98	87.16	**75.81**	79.61	**77.66**	**61.80**	67.13	**64.36**	**28.20**	77.30	**41.32**
	**SBERT**	28.44	59.50	38.22	85.37	89.57	**87.42**	73.79	76.54	75.14	60.89	63.59	62.21	26.81	74.09	39.37
	**ColBERT**	**31.19**	66.67	**42.49**	82.09	83.94	83.00	75.27	78.19	76.70	56.13	59.35	57.69	27.70	75.47	40.53
	**DPR**	28.55	60.75	38.84	**85.81**	85.40	85.60	71.82	72.74	72.28	59.00	61.74	60.34	27.16	69.37	39.23
**Llama3-70B**																
5-shot	**Base**	13.16	57.86	21.43	68.97	78.36	73.32	59.30	67.27	62.94	35.81	34.71	34.80	25.89	67.05	37.26
	**TF-IDF**	18.89	58.62	28.57	**78.49**	81.78	80.11	66.48	74.84	70.41	48.93	50.70	49.80	26.46	72.06	38.68
	**SBERT**	**23.20**	66.67	**34.42**	77.26	83.79	**80.39**	64.04	72.21	67.88	**50.66**	49.59	**50.12**	26.15	68.92	37.91
	**ColBERT**	22.05	65.12	32.94	71.21	72.33	71.76	**68.37**	75.32	**71.68**	44.93	46.08	45.50	**26.68**	72.38	**38.99**
	**DPR**	19.20	59.26	29.00	74.47	76.91	75.67	65.74	72.54	68.97	41.06	48.66	44.54	26.51	71.38	38.66
10-shot	**Base**	22.37	59.94	32.50	72.56	77.91	75.15	59.13	71.63	63.77	39.67	31.49	35.60	25.57	64.33	36.50
	**TF-IDF**	23.53	62.65	34.21	80.82	80.32	80.57	55.79	55.34	55.56	49.59	49.41	49.50	24.03	68.00	35.51
	**SBERT**	22.27	59.76	32.45	77.72	84.94	81.17	67.67	76.09	71.63	**52.84**	49.94	**51.35**	**27.61**	66.88	**39.08**
	**ColBERT**	22.58	60.50	32.89	78.40	82.37	80.34	**69.65**	76.37	**72.85**	38.72	38.81	38.77	26.49	67.58	38.06
	**DPR**	**24.37**	57.83	**34.29**	**85.16**	84.42	**84.79**	65.85	73.68	69.54	47.60	45.04	46.28	25.80	70.97	37.85
20-shot	**Base**	24.52	53.81	33.67	**75.42**	75.58	75.50	62.01	62.12	62.05	40.71	42.58	41.62	26.57	64.79	37.67
	**TF-IDF**	27.62	66.95	39.11	74.64	82.47	**78.36**	55.95	58.51	57.66	45.39	49.83	47.50	**27.80**	64.39	**38.83**
	**SBERT**	**29.93**	68.06	**41.43**	75.04	80.75	76.85	**65.90**	64.77	65.35	42.09	46.40	44.14	25.48	61.36	36.01
	**ColBERT**	23.57	65.48	34.66	73.74	70.70	72.19	58.25	57.03	57.63	**47.08**	49.88	**48.44**	25.41	66.98	36.85
	**DPR**	26.15	65.04	37.30	72.58	77.15	74.80	62.72	69.19	**65.80**	37.18	44.13	40.36	26.10	62.88	36.89

**Table 3.** Statistics of the eight standardized biomedical datasets we used, including the source and aim of their tasks, training and test sizes (number of tokens), the number of entity types and the number of entities in each dataset.

Datasets	Training Size	Test Size	Entity Types	Entities
**MIMIC III (information relating to patients)**	36.4k	6.4k	12	8.7k
**BC5CDR (extracting relationships between chemicals and diseases)**	228.8k	122.2k	2	28.8k
**Med-Mentions (annotated with UMLS concepts)**	847.9k	593.6k	1	340.9k
**NCBI Disease (PubMed abstracts annotated with disease names)**	134.0k	20.5k	4	6.3k
**Reddit-Impacts (clinical impacts and social impacts collected from Reddit)**	30.0k	6.0k	2	0.2k

## Data Availability

We utilized five distinct medical text datasets as benchmarks to evaluate the performance of our models and to support the development of new approaches. These datasets provide a diverse range of clinical narratives and biomedical information, allowing for a comprehensive assessment of our methods. The MIMIC III dataset is a large, publicly available database with patient data from critical care units, including medications, lab results, clinical notes, diagnostic codes, imaging reports, and survival data. It is widely used for few-shot classification and NER tasks. This resource extracts relationships between chemicals and diseases from annotated biomedical articles, aimed at developing systems to automatically identify these interactions for applications like drug discovery, toxicology, and understanding disease mechanisms. Med-Mentions is a large biomedical corpus annotated with UMLS concepts, containing PubMed articles linked to entities like diseases, chemicals, genes, and anatomical terms. It supports tasks such as information extraction, literature mining, and knowledge base construction. This dataset contains PubMed abstracts annotated with disease names, linked to standardized concepts in MeSH and OMIM databases. It is used to train and evaluate models for recognizing and normalizing disease names in biomedical texts. a challenging NER dataset curated from subreddits dedicated to discussions on prescription and illicit opioids, as well as medications for opioid use disorder. This dataset includes posts from 14 opioid-related subreddits, and specifically focuses on the clinical and social impacts of nonmedical substance use. Table 3 presents relevant statistics for all publicly available datasets we used in this study, including the source and aim of each dataset, training and test set sizes, the number of entity types and the number of entities in each dataset.

## Footnotes

* We excluded GPT-3.5 due to its consistent underperformance relative to GPT-4 in the previous experiments.

## Code availability

All data used in this study was publicly available. The code used for the experiments can be accessed at https://github.com/Yao-Ge-1218AM/RAG-based-Dynamic-Prompting/.
